# Evaluation of a water-based spacer fluid with additives for mud removal in well cementing operations

**DOI:** 10.1016/j.heliyon.2024.e25638

**Published:** 2024-02-13

**Authors:** Amanj Salimi, Ali Heidari Beni, Mohammad Bazvand

**Affiliations:** a Petroleum Enginnering, Sahand Oil & Gas Research Institute, Petroleum and Natural Gas Engineering Faculty of Sahand University of Technology, Iran; b Petroleum Engineering, Petroleum University of Technology, Iran

**Keywords:** Well cementation, Mud removal, Water-based spacer, Spacer fluids, Well cleaning

## Abstract

This research evaluates the crucial role of effective well cementing in enhancing petroleum production, with a specific emphasis on the utilization of spacer fluids for pre-cementing well cleaning. Investigating the performance of a water-based spacer fluid enriched with barite, Xanthan Gum, potato starch, and Poly-Anionic Cellulose additives, this study systematically designs and evaluates three distinct spacer fluids featuring varied additive concentrations for optimal mud removal efficiency. Notably, Spacer B1, incorporating 0.51% PAC-LV and 0.51% starch, emerges as the most successful, exhibiting an impressive 67.84% mud removal rate. The flow behavior of the spacers is aptly described by the Herschel-Buckley model, providing valuable insights into their rheological characteristics. Rigorous compatibility tests affirm the absence of fluid incompatibility, instilling confidence in the chosen spacer compositions. Introducing a 5% surfactant results in a noteworthy 7–8% average increase in mud removal from the metal cylinder wall. In summary, this study contributes valuable perspectives on optimizing both well cementing practices and spacer fluid formulations, ultimately elevating efficiency in petroleum production processes.

## 1 Introduction

Well cementing is a critical part of well completion operations as it ensures well integrity and prevents potential hazards such as blowouts. Cement slurry is pumped behind the casing to isolate different zones of the well, prevent communication between various fluids, prevent casing corrosion, and avoid excessive water production and gas migration. Despite the progress made in the oil industry, the cementing operation remains relatively unchanged since its inception in 1903. One of the significant challenges in cementing is the removal of drilling fluid from the annular space during cementing. This task is vital for effective isolation of hydrocarbon production areas throughout the life of the reservoir. Removing drilling fluid can be problematic due to its incompatibility with cement, causing a gel-like state at the mud and cement's contact surface, leading to clogs and cracks in weak formations [[1], [2], [3]].

To address this issue, efforts have been made to eliminate all drilling fluids from the annular space. The use of spacer fluid or pre-flush fluid, or both fluids, has been suggested to remove the drilling fluid before cementing. Spacer fluid design should rely on well-provided data to ensure optimal hydrocarbon production areas are isolated. Proper clearance of drilling mud and effective displacement of fluids inside the well are critical to developing successful cement bondings and avoiding communication issues between zones. To achieve these objectives, spacers include various additives such as weighting agents, polymers, and surfactant. In summary, the use of spacer fluids in cementing operations is crucial to achieving proper clearance of drilling mud and effective displacement of fluids inside the well. The composition ratio of the additives used in spacers plays a vital role in achieving the desired properties in the fluids. However, cement contamination by other fluids is a significant challenge that reduces the success rate of the cementing operation [4]. To address this challenge, in a recent study, Bois et al. [5] have developed new spacer fluids, such as foam-based and insulating spacer fluids, to remove drilling fluids and reduce filter cakes. Additionally, Siddig et al. [6] have suggested nano-emulsion technology to change the surface wettability of the formation, which can increase the success rate of cementing operations. Cia et al. (2022) have shown that proper planning and execution of the cementing operation can also have a significant impact on the success rate. This involves the selection of an appropriate type of cement, designing the cement slurry, and ensuring proper placement by centering the casing in the wellbore. By analyzing effective parameters and simulating multi-fluid flows, the amount of cement contamination by the spacer and drilling fluid can be optimized. Moreover, proper planning and execution of the cementing operation can also have a significant impact on the success rate. This involves selecting the appropriate type of cement, designing the cement slurry to suit the wellbore conditions, and ensuring proper placement of the cement within the well. The use of centralizers can help achieve proper placement by centering the casing in the wellbore and creating an annular space for the cement slurry [3,7]. Furthermore, during the cementing operation, monitoring the cement placement and verifying the integrity of the cement sheath is essential. This can be done using various techniques, including temperature logging, pressure testing, and cement evaluation tools such as ultrasonic imaging ([[8], [9], [10]]; Halliburton & Schlumberger, 2023).

Elochukwu et al. [11] investigated the efficacy of a methyl ester sulphonate (MES) spacer fluid additive for optimizing wellbore clean-up, proposing an environmentally-friendly alternative to conventional oil-based drilling fluids. The research explores MES's impact on water-based drilling fluid rheology, cement bonding in permeable pay zones, and its environmental footprint compared to oil-based drilling fluids. Experimental assessments cover MES spacer fluid rheology, emulsion disruption, wettability changes on steel and sandstone, and compatibility with oil-based drilling fluid and cement. The study also evaluates MES spacer fluid cleaning efficiency and shear bond strength. Contact angle measurements, conducted with a drop shape analyzer and high-definition camera, determine wettability changes induced by oil-based drilling fluid (OBDF) and MES spacer fluid on steel and sandstone surfaces. A range of 0°–90° indicates a water-wet state, while angles above 90° suggest an oil-wet state, providing valuable insights into OBDF and MES spacer fluid wettability alterations [11].

In conclusion, the success of cementing operations depends on various factors, including the design of spacer fluids, proper planning and execution of the operation, and monitoring the placement and integrity of the cement sheath. Identifying these factors and implementing appropriate measures can significantly improve the success rate of cementing operations and reduce the potential for wellbore failures. In this paper, the structure was defined to provide a clear and concise presentation of the information. The paper began with an introduction to the importance of well cementing in petroleum production and the use of spacer fluids to clean the well before cementing. It then focused on the design and evaluation of a water-based spacer fluid with barite, Xanthan Gum, potato starch, and Poly-Anionic Cellulose as additives for mud removal. In the methodology section, we discussed the selection of the Herschel-Buckley model to describe the flow behavior of the spacers and the results of compatibility tests. The results section highlighted the effect of adding surfactant on mud removal. Finally, the paper concluded by summarizing the key findings and emphasizing the importance of optimizing well cementing and spacer fluid composition to improve petroleum production efficiency.

## 2 Materials and experimental methods

In this section materials and methodology is presented in order. The study employed a comprehensive set of experimental methods and testing procedures to assess the performance of the designed spacer fluids in drilling applications. Rheological tests, including the Bingham Plastic (BP) model, Power Law model, and Herschel-Buckley model, were conducted to characterize the flow behavior of spacer fluids, elucidating the relationship between shear stress and shear rate in non-Newtonian fluids. Viscometer experiments at different speeds (3, 6, 100, 200, 300, and 600 RPM) provided a detailed understanding of how spacer fluids respond to varying shear rates, aiding in determining their suitability under diverse drilling conditions. The rotor test evaluated the practical cleaning efficiency of spacer fluids by assessing their ability to clean drilling fluid from the metal wall of a cylinder, offering valuable insights into maintaining a clean drilling environment. The mud removal test quantified the efficiency of spacer fluid in cleaning drilling fluid from the metal cylinder wall, providing a quantitative measure of cleaning capabilities. Fluid loss control tests assessed the impact of different additives on spacer fluids' filter loss, crucial for preventing wellbore instability. Compatibility tests investigated the rheological properties of spacer fluids in contact with drilling fluid and cement slurry, identifying potential issues related to fluid compatibility. In summary, these chosen experimental methods and testing procedures collectively contribute to a comprehensive evaluation of spacer fluids, supplying valuable data for optimizing their composition and performance in drilling operations.

### 2.1 Cement slurry

G class cement is used to design the cement slurry, because this type of cement is often used in most wells in Iran.

### 2.2 Drilling fluid composition

The drilling fluid selected in this study is bentonite and the appropriate concentration of bentonite in this type of drilling fluid is equal to 20 lb/bbl acoording to reference (Deshpande, 2016).

The characterization of the designed cement slurry, composition and properties of bentonite drilling fluid (which is designed in this study) are given in Table 1 and Table 2.

**Table 1:** Properties and compounds of cement slurry.

Characterizations	Type/Value
**Cement class**	**G**
**Water/Cement Ratio**	**46.0**
**Density**	**pcf119**
**Plastic viscosity**	**cp150**
**Yield Point**	**Lb/100ft**^**2**^**10**

**Table 2:** Properties and compositions of designed drilling fluid.

Composition		Concentration)lb/bbl)
Soda Ash		0.2
NaOH		0.5
KCl		3
Starch		4.5
PAC-LV		4
Bentonite		20
Density		65.1 pcf
PH		12.42
Primary Fluid loss		2 ml
Fluid Loss after 30 min		5 ml
Plastic Viscosity		13 cp
Mud Cake Thickness(ml)		55
GS(10 s) (lb/100 ft^2^)	3RPM	5
	6RPM	7

After conducting multiple tests to design the drilling fluid, the optimal concentration of starch, bentonite, and polymers was determined, resulting in the reduction of fluid loss from 20 ml in the initial test to only 5 ml in the final test, each lasting 30 min. In all experiments conducted in the development of WBM, KCl, NaOH, and Soda Ash remained constant at 0.79%, 0.13%, and 0.05%, respectively. Once the quantity of these three additives was stabilized, the influence of three other additives, namely bentonite, PAC-LV, and starch, on filter loss was tested in the drilling fluid composition. The step-by-step tests for the design of the water-based drilling fluid are outlined in Table 3.

**Table 3:** The weight percentage of various additives used in drilling fluid to optimize their concentrations.

Number of test	%weight Bentonite	%weight Starch	%weight PAC	%weight KCL	%weight NaOH	%weight Soda Ash	Fluid Loss (ml/30 in)
**1**	**3.98**	**1.19**	**1.06**	**0.79**	**0.13**	**0.05**	**10**
**2**	**5.23**	**1.18**	**1.05**	**0.78**	**0.13**	**0.05**	**5**
**3**	**6.46**	**1.16**	**1.03**	**0.77**	**0.13**	**0.05**	**6**
**4**	**5.27**	**0.4**	**1.05**	**0.79**	**0.13**	**0.05**	**14**
**5**	**5.25**	**0.79**	**1.05**	**0.79**	**0.13**	**0.05**	**8**
**6**	**5.23**	**1.18**	**1.05**	**0.78**	**0.13**	**0.05**	**5**
**7**	**5.28**	**0.79**	**0.53**	**0.79**	**0.13**	**0.05**	**12**
**8**	**5.27**	**0.79**	**0.79**	**0.79**	**0.13**	**0.05**	**10**
**9**	**5.25**	**0.79**	**1.05**	**0.79**	**0.13**	**0.05**	**8**

**Fig. 1:**
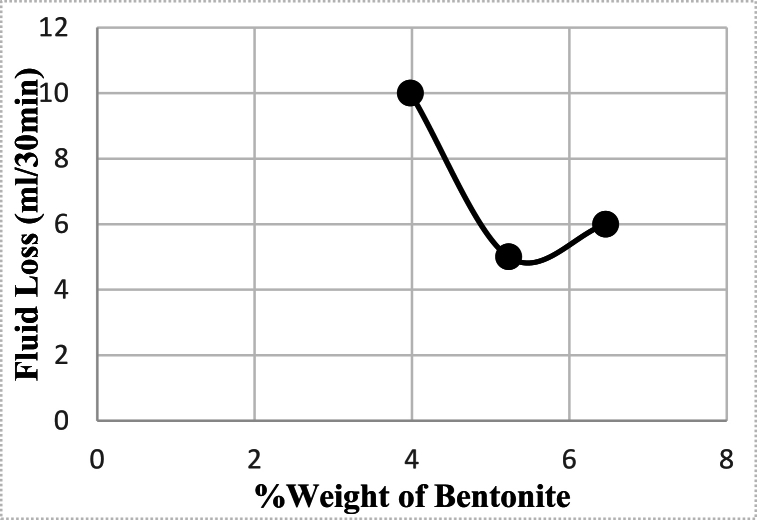
The effect of bentonite concentration on the Filter loss after 30 min (containing 3 g starch and 4g PAC-LV).

Table 3 presents experiments 1, 2, and 3, where all additives, except bentonite, were held constant, and bentonite was the only variable parameter. The optimal concentration for bentonite was found to be 5.23% by weight of fluid or 20 lb/bbl. At this concentration, the rate of filtration loss was minimized, and at different concentrations, the volume of filtrate increased, as illustrated in Fig. 1.

**Fig. 2:**
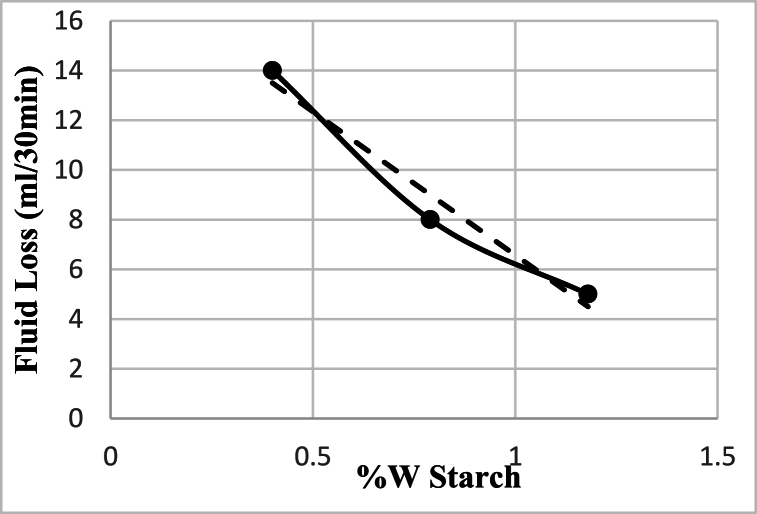
The effect of starch concentration on the Filter loss after 30 min (containing 20g bentonite and 4g PAC-LV).

Furthermore, the impact of starch concentration on the volume of filtrate of drilling fluid was examined. Three different weights of starch were added, while all other additives were used in fixed amounts in the fluid. It was observed that as the starch concentration increased, the volume of filtrate decreased. The relationship between starch concentration and volume of filtrate is depicted in Fig. 2, where a linear trend line equation (equation 1) was obtained from the first degree. The equation is provided below:

Y = −11.54X+18.12 (1)

R^2^ = 0.9643.

**Fig. 3:**
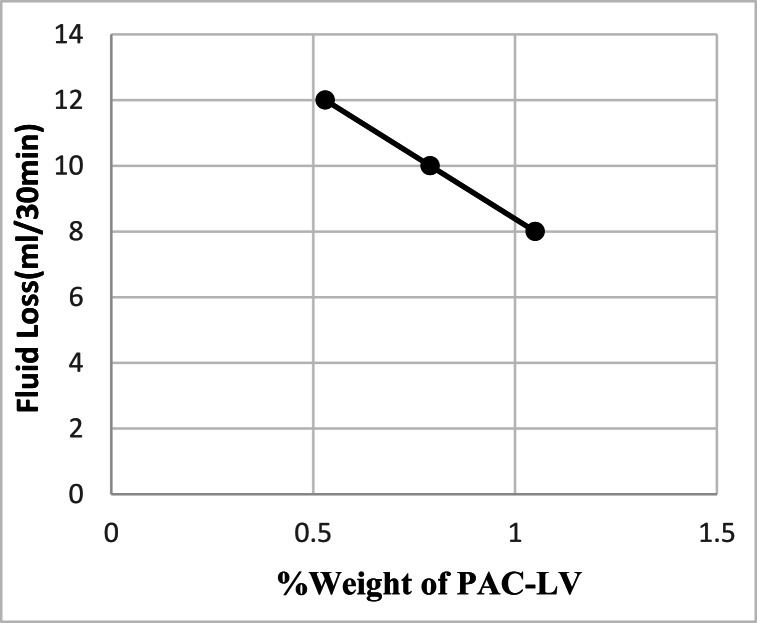
The effect of PAC-LV concentration on the Filter loss after 30 min (containing 20g bentonite and 4g starch).

When too much starch is added to the drilling fluid, it can absorb water molecules and create a highly viscous fluid that cannot be easily pumped into the well. Similarly, excessive PAC-LV concentration can also cause an increase in viscosity and impede the ability of fluid to be pumped into the well. To investigate the effect of PAC-LV concentration on fluid loss, the filtrate was tested and was found to decrease linearly with an increase in PAC-LV concentration. Fig. 3 illustrates the changes in the volume of filtrate with respect to PAC-LV concentrations, with the trend line equation shown (equation (2)).

(2) Y = −7.69X+16.08

R^2^ = 1

**Fig. 4:**
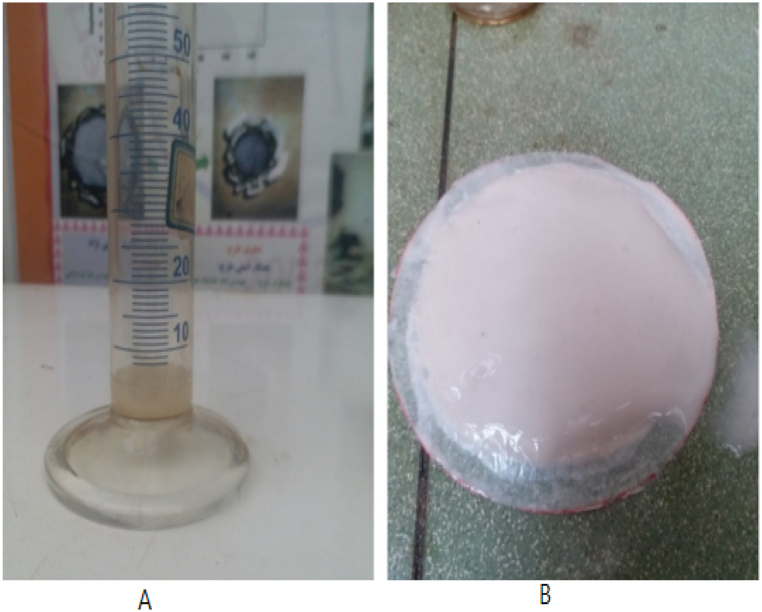
A) The obtaining volume of filtrate after 30 min. B) The thickness of the mud cake formed is shown on filter paper.

Fig. 4a and b shows an example of a filtration drop test for the drilling fluid sample used in this research.

### 2.3 Spacer designing

The following properties are essential for each spacer.

1) Compatibility with drilling fluid and cement slurry
2) Controlled viscosity for creating a plug flow to move drilling fluids out of the well
3) Pumpability with cementing equipment
4) Ability to carry cuttings ([12]; foroushan et al., 2020)
5) Capability to wash the mud cake from the formation (Richardson et al., 2013)
6) Conversion of the formation surface from oil-wet to water-wet (in the case of using Oil Based Mud (OBM)) ([13]; Hunt et al., 1982)
7) Cost-effectiveness

In order to achieve the aforementioned objectives, various additives need to be incorporated into the water-based base fluid used in this study. These additives include the following.

1) Weighting Agent: Barite was utilized as the weighting agent to increase the weight of the spacer to the desired amount, which is the weight of the spacer between the drilling fluid and the cement slurry.
2) Viscosifier: XC was used as the viscosifier to control the viscosity of the spacer.
3) Lost Circulation Material: To prevent fluid loss, a combination of potato starch and PAC-LV were used.
4) pH Controller: The pH level of the fluid should be between 9 and 10. Soda ash and caustic soda were used to regulate the pH of the fluid, and their values were kept constant throughout all experiments. Soda ash also has the added function of depositing calcium ions in water.

The optimal concentrations of additives within the spacers were determined through a systematic and comprehensive experimental approach. We conducted a series of experiments varying the concentrations of each additive, including weighting agents, polymers, and surfactants, individually. These experiments involved assessing the performance of the spacer in terms of shear-bond strength, rheological properties, and cleaning efficiency under different conditions. The concentration ranges were carefully chosen based on the observed effects on spacer performance, ensuring that we explored a spectrum of concentrations to identify the most effective and efficient formulations.

Three types of spacers were created with varying proportions to correspond with the drilling fluid used. Barite was used as the weighting agent in all selected fluids and was designed based on three distinct weights: 72 lb/ft3, 82 lb/ft3, and 95 lb/ft3. The components and characteristics of all three designed spacers are presented in Table 4, Table 5, Table 6.

**Table 4:** Composition and specification of spacer A

		Spacer A		
		**A**_**1**_	**A**_**2**_	**A**_**3**_
**Soda ash(lb/bbl)**		**2.0**	**2.0**	**2.0**
**Naoh(lb/bbl)**		**5.0**	**5.0**	**5.0**
**barite(lb/bbl)**		**35**	**59**	**114**
**Starch(lb/bbl)**		**-**	**-**	**-**
**PAC-LV(lb/bbl)**		**3**	**5.3**	**4**
**XC(lb/bbl)**		**2**	**2**	**2**
**Density(pcf)**		**69**	**80**	**93**
pH		**9.5**	**9.3**	**9.1**
**Fluid Loss after 30 min**		**13**	**11**	**10**
**Plastic Viscosity**		**19**	**20**	**29**
**GS (10 s) (ft**^**2**^**100 lb/)**	**3 RPM**	**29**	**43**	**94**
	**RPM6**	**31**	**45**	**101**

**Table 5:** Composition and specification of spacer B.

		Spacer B		
		**B**_**1**_	**B**_**2**_	**B**_**3**_
**Soda ash(lb/bbl)**		**2.0**	**0.2**	**0.2**
**Naoh (lb/bbl)**		**5.0**	**5.0**	**5.0**
**Barite(lb/bbl)**		**35**	**59**	**114**
**Starch(lb/bbl)**		**2**	**5.2**	**5.3**
**PAC-LV(lb/bbl)**		**2**	**5.2**	**3**
**XC(lb/bbl)**		**-**	**-**	**-**
**Density(pcf)**		**72**	**80**	**95**
pH		**5.9**	**4.9**	**2.9**
**Fluid Loss after 30 min**		**15**	**11**	**5**
**Plastic Viscosity**		**22**	**18**	**26**
**GS (10 s) (ft**^**2**^**100 lb/)**	**3RPM**	**23**	**36**	**59**
	**RPM6**	**24**	**38**	**59**

**Table 6:** Composition and specification of spacer C.

		Spacer C		
		**C**_**1**_	**C**_**2**_	**C**_**3**_
**Soda ash(lb/bbl)**		**2.0**	**0.2**	**0.2**
**Naoh(lb/bbl)**		**5.0**	**5.0**	**5.0**
**Barite(lb/bbl)**		**35**	**59**	**114**
**Starch(lb/bbl)**		**5.2**	**25.3**	**4**
**PAC-LV(lb/bbl)**		**-**	**-**	**-**
**XC(lb/bbl)**		**2**	**2**	**2**
**Density(pcf)**		**71**	**80**	**92**
pH		**.69**	**5.9**	**.29**
**Fluid Loss after 30 min**		**14**	**11**	**9**
**Plastic Viscosity**		**17**	**16**	**17**
**GS (10 s) (ft**^**2**^**100 lb/)**	**3RPM**	**19**	**18**	**20**
	**RPM6**	**20**	**19**	**20**

**Fig. 5:**
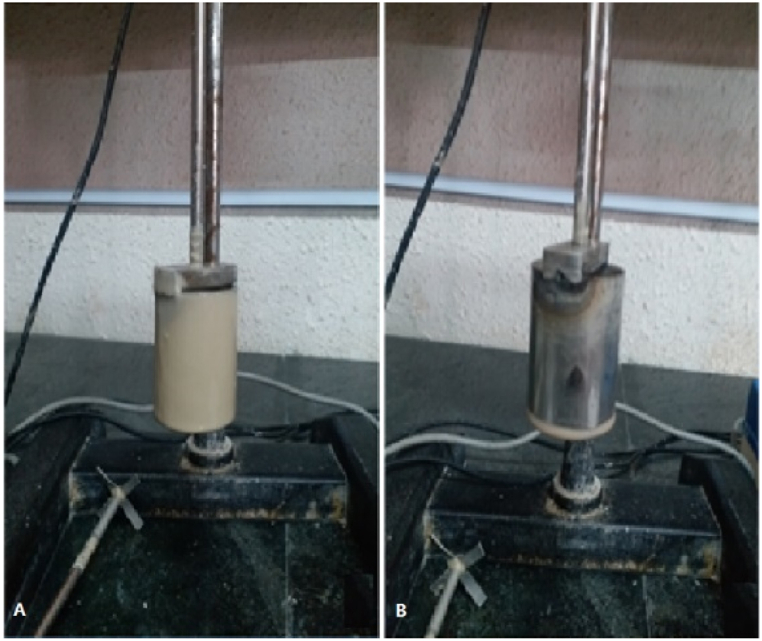
Example of performing a test rotor test; A) forming a mud cake on the surface of the cylinder after rotation in the drilling mud. B) cleaning the surface of the cylinder after rotation in the spacer A2.

Based on Table 4, Table 5, Table 6, all three spacers showed acceptable fluid loss with a filtrate volume consistently below 20 ml/30min across various LCM concentrations. Spacer B, however, exhibited better performance overall compared to Spacer A and C. Spacer A and C contained XC polymer, resulting in greater gel resistance due to the formation of longer polymer chains and wide lattice structure, with PAC-LV having a greater effect than starch in increasing gel resistance. Spacer A had the highest gel resistance while Spacer B had the lowest. Additionally, XC had a stronger effect on plastic viscosity than PAC-LV and starch due to its high ionic properties, resulting in Spacer A having the highest plastic viscosity and Spacer B having the lowest. For ease of calculation and further sample design information, Table 7 provides the weight percentage of all additives used in the spacers. In order to assess the cleaning efficiency of the spacers, a test rotor test was performed, as illustrated in Fig. 5. Panel A of the figure shows the formation of a mud cake on the surface of the cylinder after rotation in the drilling mud. Panel B demonstrates the cleaning process of the surface of the cylinder after rotation in Spacer A2.

**Table 7:** %Weight of different additives in 9 samples of spacer fluid designed.

Sample	%weight Soda Ash	%weight NaOH	%weight XC	%weight PAC-LV	%weight Starch	%weight Barite
**A**_**1**_	**0.05**	**0.13**	**0.51**	**0.77**	**0**	**8.96**
**A**_**2**_	**0.05**	**0.12**	**0.48**	**0.84**	**0**	**14.21**
**A**_**3**_	**0.04**	**0.11**	**0.42**	**0.85**	**0**	**24.22**
**B**_**1**_	**0.05**	**0.13**	**0**	**0.51**	**0.51**	**8.98**
**B**_**2**_	**0.05**	**0.12**	**0**	**0.60**	**0.60**	**14.23**
**B**_**3**_	**0.04**	**0.11**	**0**	**0.64**	**0.74**	**24.19**
**C**_**1**_	**0.05**	**0.13**	**0.51**	**0**	**0.64**	**8.97**
**C**_**2**_	**0.05**	**0.12**	**0.48**	**0**	**0.78**	**14.22**
**C**_**3**_	**0.04**	**0.11**	**0.42**	**0**	**0.85**	**24.22**

### 2.4 Rheological models

Rheological models have been developed to describe the relationship between shear stress and shear rate in non-Newtonian fluids. The following is the description of these models:

**BP model.** The most important difference between this model and the Newtonian fluid model is the presence of yield stress. As long as the force does not overcome the yield stress, there is no volumetric motion in the fluid, and after overcoming this yield stress, the shear stress relative to the shear rate can be changed. Bingham's plastic which states as equation (3) is as follows (Zanten et al., 2010):

(3) $$\tau =Y_{p}+\mu_{p}\times \gamma \text{,}$$

Where;

τ is Shear Stress.

$Y_{p}$ = Yield point

$\mu_{p}$ = Plastic Viscosity

γ = Shear Rate

**Power law Model.** The BP model has some defects, and the power law emerged to solve them. In this model, changes in shear stress are proportional to changes in shear rate. This model is graphically between Newton and BP models and is used to describe pseudo-plastic fluid. The equation of the power law model is as follow (Zanten et al., 2010):

(4) $$\tau =K\;\left(\gamma^{n}\right)\text{,}$$

τ = Shear Stress

K = the Flow Consistency Index.

γ = Shear Rate

n = Power Law constant.

In equation (4), the power n and the coefficient K for each fluid are constant and different from other fluids. The coefficient k is called the fluid stability index and shows the pump-ability of the fluid. As the value of k increases, the viscosity of the fluid increases and it becomes more difficult to pump. Power n indicates the degree of deviation of a non-Newtonian fluid from a Newtonian fluid. The power n for Newtonian fluid is equal to one (Drilling Fluid Reference Manual, 2006).

**Pseudo-Plastic Fluids.** In this fluid model, the constant power factor n < 1 and with increasing shear stress, the apparent viscosity increases which is shown in equation (5).

(5) $$SS=K{\left(SR\right)}^{n},\ldots \ldots \ldots \ldots \ldots \ldots \ldots \ldots \ldots ..\ldots \ldots \ldots \ldots .\;n<1,\ldots .\ldots \ldots \ldots \ldots \ldots \ldots \ldots \ldots \ldots \ldots \ldots \ldots \ldots \ldots \ldots \ldots \ldots$$

SS=Shear Stress.

SR=Shear Rate.

**Dilatant Fluids.** In this fluid model, the constant power factor n > 1 and the dilatant fluid equation (6) is as follows:

(6) $$SS=K{\left(SR\right)}^{n},\ldots \ldots \ldots \ldots \ldots \ldots \ldots \ldots \ldots ..\ldots \ldots \ldots \ldots .\;n>1,\ldots \ldots \ldots \ldots \ldots \ldots \ldots \ldots \ldots \ldots \ldots \ldots \ldots \ldots \ldots \ldots .\ldots \ldots$$

**Herschel-Buckley model.** The HB equation performs better than the power model and the BP model because in this model the yield stress is used in the equation's formula. The HB model equations (7), (8) is given below:

(7) $$\tau {=\tau }_{0}+k\gamma^{n}\text{,}$$

(8) $$\log\left(\tau -\tau_{0}\right)=\log\left(k\right)+n\times \log\;\left(\gamma \right)\text{,}$$

The yield stress is normally considered to be the same as that obtained at 3 RPM. However, in this article it is calculated according to the method of Versan, M. and Tolga (2005). and placed in equation (9). The value of τ_0_ is calculated through Equation (7):

(9) $$\tau_{0}=\frac{\tau^{*2}-\tau_{\min}\times \tau_{\max}}{2\times \tau^{*}-\tau_{\min}-\tau_{\max}}\text{,}$$

Here, $\tau^{*}$ is the shear stress corresponds to the geometric mean shear rate ($\gamma^{*}$) which can be obtained by relation (8) and shown in equation (10):

(10) $$\gamma^{*}=\sqrt{\gamma_{\min}\times \gamma_{\max}}\text{,}$$

Shear rate was constant in all experiments, so the average shear rate is always the same and equals to $\gamma^{*}$ = 72.25. To obtain the shear stress at this average shear rate, it is sufficient to perform an interpolation and calculate $\tau^{*}$. Then put the obtained value of $\tau^{*}$ in equation (9) and the yield stress value will be obtained.

After obtaining the yield stress, the value of n and k should be calculated. One of the ways to remember the two parameters n and k is to use the deviation angle obtained at the rotational speeds of 300 RPM and 600 RPM. Another way to calculate them is to use a graph, in this way, the value of $\tau -\tau_{0}$ relative to γ is plotted in a logarithmic graph and linear regression is taken and an equation is formed. The value of k is obtained from the constant number and the value of n is the power of this equation. In the following figure, an example of the graph and the value of n and k is given.

**Fig. 6:**
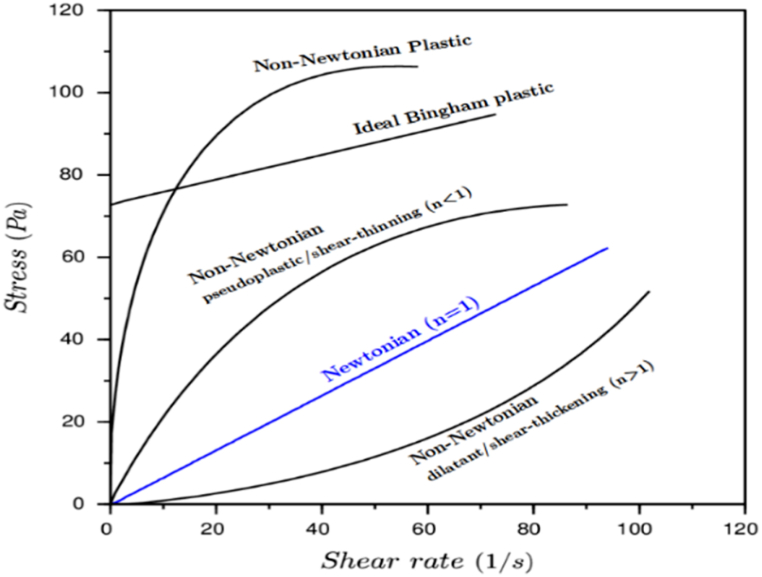
Fluid flow behavior curves according to different rheological models for non-Newtonian fluids.

**Fig. 7:**
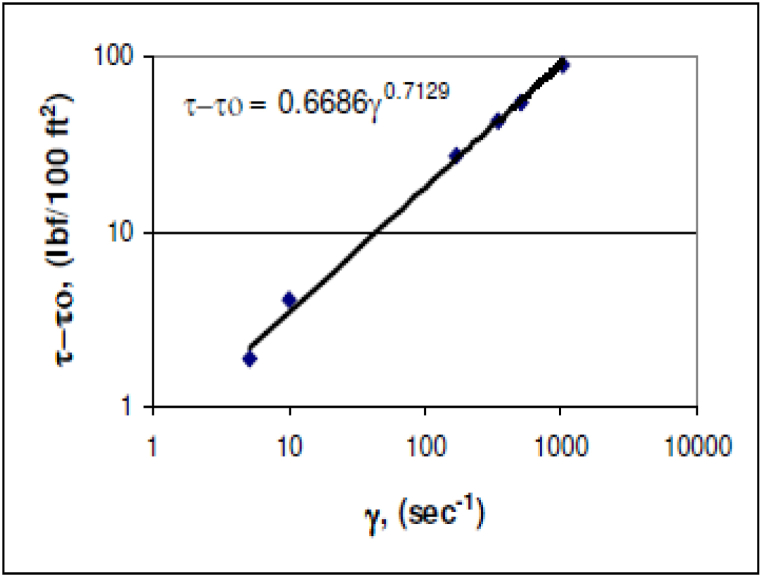
Hb fluid rheogram.

Fig. 6 provides an illustrative example of fluid flow behavior curves according to different rheological models for non-Newtonian fluids, showcasing the values of n and k obtained through this method. Acoording to Fig. 7, n = 0.7129.

The power n in the equation is one of the factors affecting drilling particles and if the value of n decreases, it means that the carrying capacity of drilling particles is reduced and with increasing the shear rate, the viscosity of fluids with a low value of n increases [14].

All 9 different models of spacers at speeds of 3, 6, 100, 200, 300 and 600 RPM were tested in the viscometer and the deflection angle obtained in these tests by the spacers are shown in Table 8.

**Table 8:** Rheological data obtained for spacers in viscometer experiments.

**Velocity (RPM)**		Spacer A			Spacer B			Spacer C		
		**A**_**1**_	**A**_**2**_	**A**_**3**_	**B**_**1**_	**B**_**2**_	**B**_**3**_	**C**_**1**_	**C**_**2**_	**C**_**3**_
**3**		**17**	**20**	**29**	**13**	**14**	**15**	**10**	**17**	**18**
**6**		**19**	**24**	**37**	**17**	**18**	**18**	**12**	**20**	**26**
**100**		**33**	**45**	**93**	**27**	**32**	**42**	**28**	**44**	**50**
**200**		**45**	**61**	**111**	**37**	**41**	**52**	**39**	**61**	**75**
**300**		**55**	**79**	**124**	**45**	**51**	**61**	**54**	**72**	**102**
**600**		**81**	**110**	**150**	**70**	**75**	**80**	**73**	**93**	**129**
**GS (10sec)** **(lb/100 ft**^**2**^**)**	**3RPM**	**29**	**43**	**94**	**19**	**18**	**20**	**23**	**36**	**59**
	**6RPM**	**31**	**45**	**101**	**20**	**19**	**20**	**24**	**38**	**59**
**GS (10min)** **(lb/100 ft**^**2**^**)**	**3RPM**	**30**	**42**	**98**	**19**	**19**	**22**	**25**	**36**	**61**
	**6RPM**	**33**	**49**	**106**	**21**	**20**	**22**	**25**	**37**	**64**
**PV(lb/100 ft**^**2**^**)**		**26**	**31**	**26**	**25**	**24**	**19**	**19**	**21**	**27**
**YP (lb/100 ft**^**2**^**)**		**29**	**48**	**98**	**20**	**27**	**42**	**35**	**51**	**75**
**YP/PV Ratio**		**1.12**	**1.55**	**3.77**	**0.80**	**1.13**	**2.21**	**1.84**	**2.43**	**2.78**
**n (HB model)**		**0.6**	**0.58**	**0.42**	**0.6**	**0.58**	**0.54**	**0.69**	**0.61**	**0.59**
**K (HB model)**		**0.85**	**1.72**	**8.39**	**0.88**	**1.18**	**1.86**	**0.59**	**1.33**	**2.19**

The YP/PV ratio is a critical parameter used to evaluate the stability of drilling fluids. A higher YP/PV ratio indicates better suspension ability of solid particles and higher fluid stability. However, increasing this ratio may make it more challenging to pump fluids into the well due to the increased yield point and gel resistance. The acceptable range of YP/PV for drilling fluids is between 1.5 and 3. As shown in Table 8, all spacers except A3 fall within this range. Spacer A has a slightly higher YP/PV ratio due to the presence of PAC-LV in its composition, which prevents settling of barite and solid particles. Additionally, the XC polymer in spacer A increases the yield stress and YP/PV ratio, resulting in a higher value than spacer B. The parameter K indicates the stability of the spacer, and a higher value indicates better stability. Spacer A has a higher K value, indicating higher viscosity and potential difficulty in pumping into the well. However, the use of PAC-LV polymer and XC polymer in its composition increases its stability. Spacer C has an acceptable YP/PV ratio and a relatively stable K value, indicating an acceptable ability to pump fluids into the well and spacer stability due to the presence of XC polymer. Spacer B has a lower YP/PV ratio than the other two spacers due to the absence of XC polymer in its composition, which increases the YP/PV ratio and suspension ability of solid particles due to increased spacer viscosity (Viloria et al., 2006).

## 3 Results and discussion

### 3.1 Comparison of results from rheological data results

**Fig. 8:**
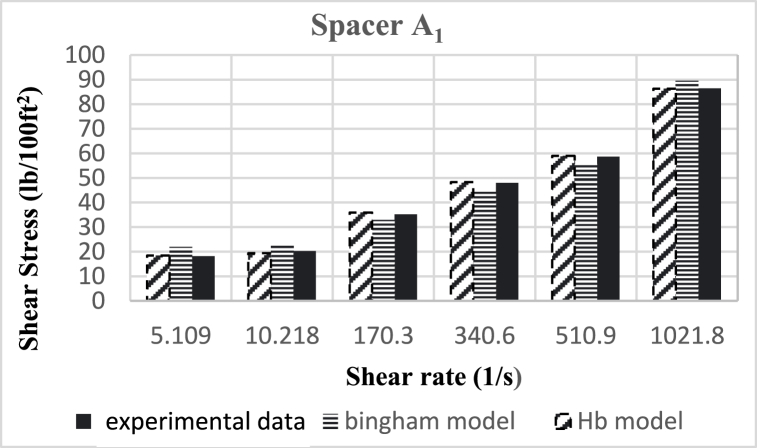
Comparison diagram of different rheology models to investigate the behavior of spacer A1.

**Fig. 9:**
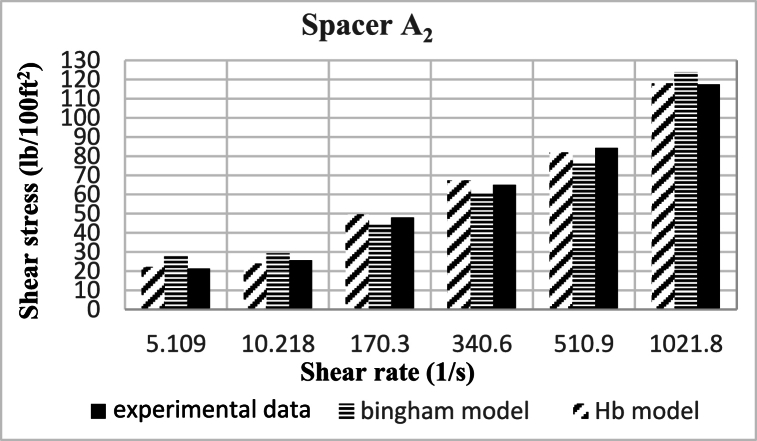
Comparison diagram of different rheology models to investigate the behavior of spacer A2.

**Fig. 10:**
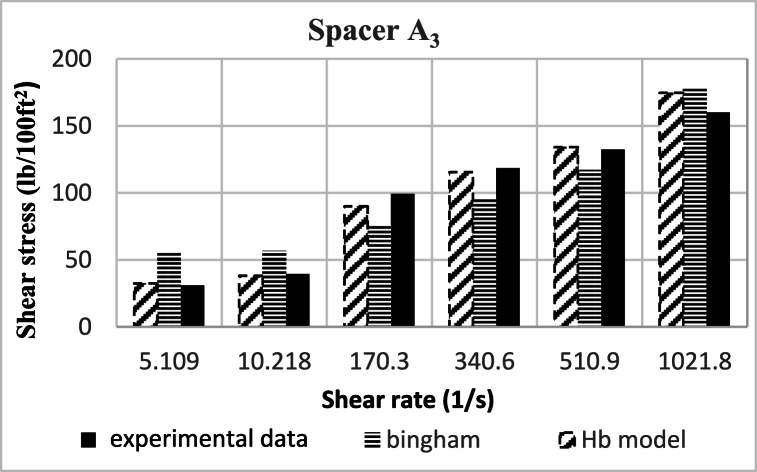
Comparison diagram of different rheology models to investigate the behavior of spacer A1.

**Fig. 11:**
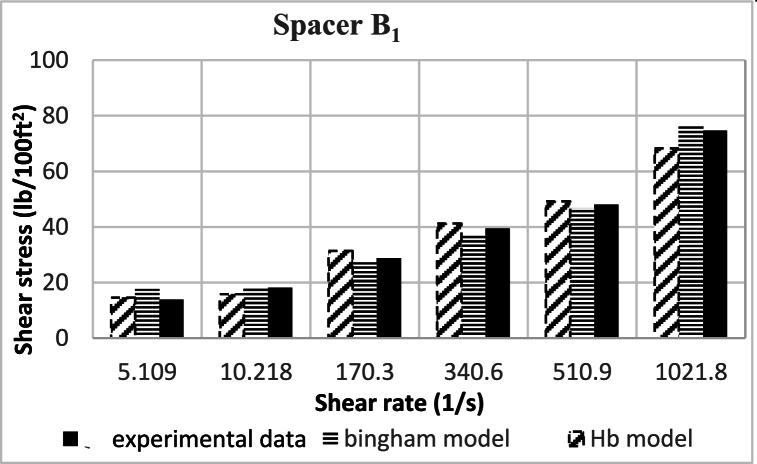
Comparison diagram of different rheology models to investigate the behavior of spacer B1.

**Fig. 12:**
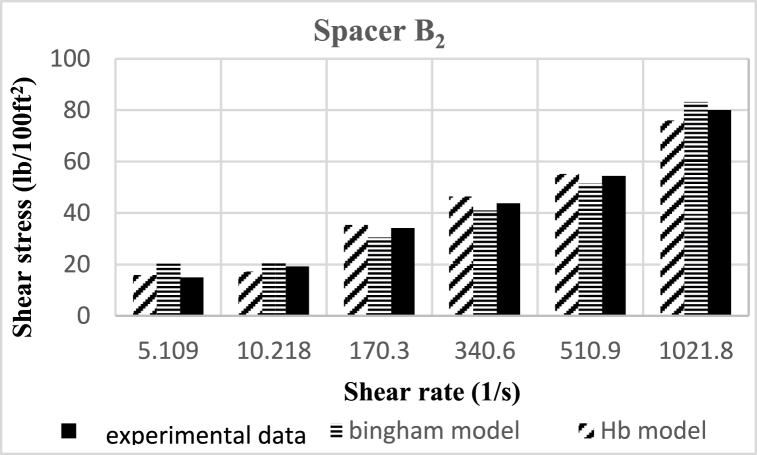
-Comparison diagram of different rheology models to investigate the behavior of spacer B2.

**Fig. 13:**
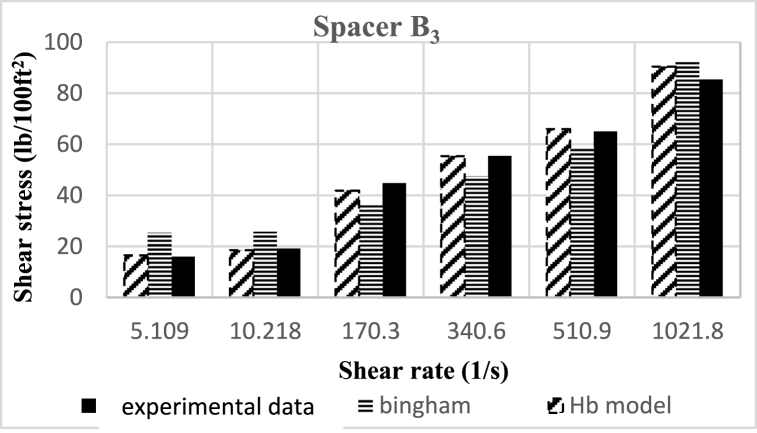
Comparison diagram of different rheology models to investigate the behavior of spacer B3.

**Fig. 14:**
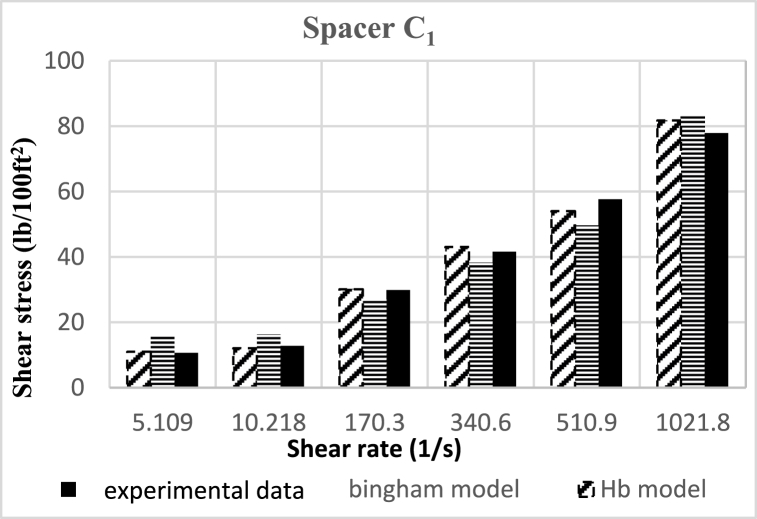
Comparison diagram of different rheology models to investigate the behavior of spacer C1.

**Fig. 15:**
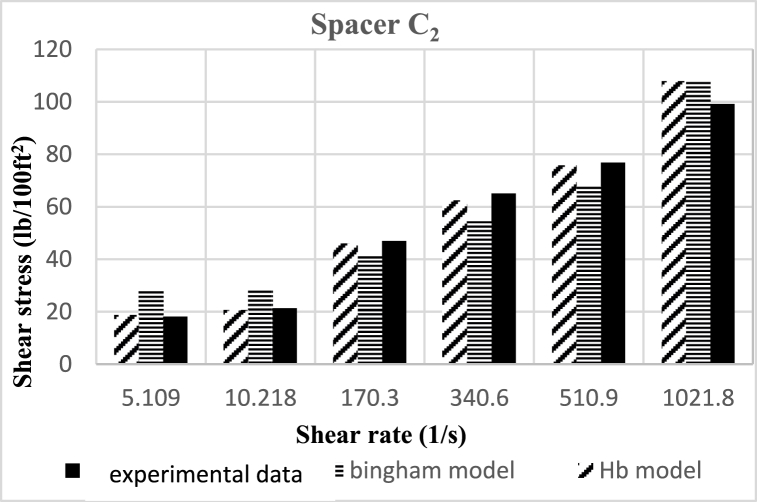
Comparison diagram of different rheology models to investigate the behavior of spacer C2

**Fig. 16:**
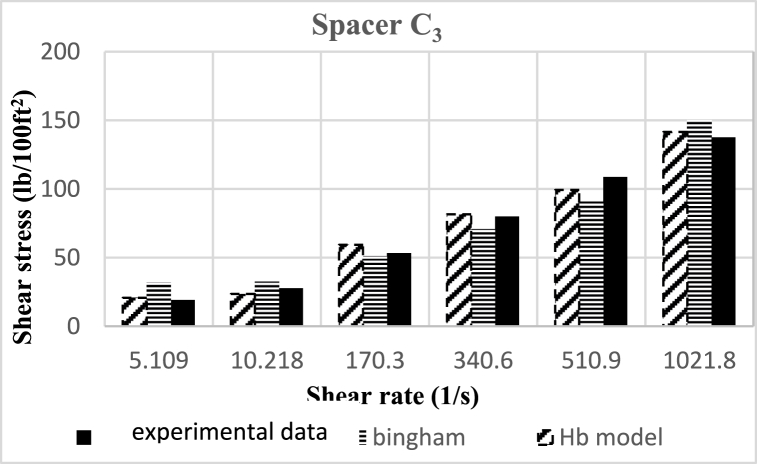
Comparison diagram of different rheology models to investigate the behavior of spacer C3.

**Fig. 17:**
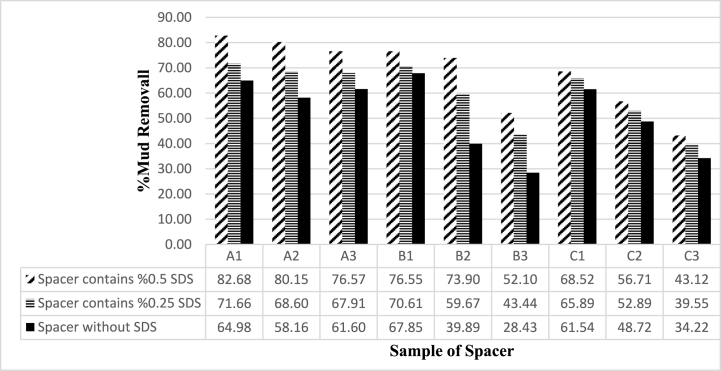
Effect of spacer fluid on mud removal in two cases without using SDS surfactant and using SDS surfactant.

Based on research, the spacers designed in this study align better with the Herschel-Buckley (HB) model. The fluids were evaluated using both HB and Bingham Plastic (BP) models, and by comparing the experimental data with the results obtained from the two models, it was concluded that the HB model is more consistent with the experimental data, providing a better description of the studied spacer fluids. Fig. 8, Fig. 9, Fig. 10, Fig. 11, Fig. 12, Fig. 13, Fig. 14, Fig. 15, Fig. 16 were generated based on these results and plotted in terms of shear stress relative to the shear rate of the viscometer. Comparing the two models, it is evident that the HB model is more consistent with the experimental data. In a few experiments, an increase in error percentage of the HB model was observed, which could be attributed to human error or laboratory issues such as machine or calculation errors. Overall, the average error percentage of the HB model for the seven spacer fluid samples was less than 6%, with only 7% error obtained for two spacer fluid samples (see Fig. 17).

This study intricately explores the intrinsic mechanisms dictating the rheological behavior of spacer fluids, specifically focusing on Spacer A, which comprises XC polymer, PAC-LV, and starch. The noteworthy rheological modeling in this paper lays the groundwork, prompting a response to the insightful suggestion by the reviewer to elucidate micro-level interactions and mechanisms, especially those involving starch. Spacer A's composition incorporates XC polymer, PAC-LV, and starch, each contributing distinct characteristics. Understanding the individual roles of these components is pivotal for deciphering the intricate rheological responses observed during experiments. Within Spacer A, XC polymer plays a pivotal role in forming elongated polymer chains and a wide lattice structure, thereby contributing to enhanced gel resistance and increased plastic viscosity. PAC-LV collaborates with XC polymer, amplifying gel resistance and significantly contributing to overall viscosity. The synergistic effect of PAC-LV with starch elevates the fluid's ability to resist gel formation. Starch, while independently contributing to gel resistance, also plays a crucial role in controlling fluid loss. The micro-level interactions among XC polymer, PAC-LV, and starch result in a well-balanced rheological profile, ensuring an optimal combination of gel resistance, plastic viscosity, and fluid loss control. Although plots are not included, this textual elucidation aims to provide readers with a profound comprehension of the intricate micro-level mechanisms shaping the macroscopic rheological behavior of Spacer A. The molecular interplay among these admixtures determines the overall performance of the spacer fluid, aligning with the objectives of this study to enhance understanding of designed spacer fluids for drilling applications. This detailed examination of Spacer A's intrinsic mechanisms seeks to enhance the overall understanding of its rheological properties. While the absence of plots may limit the visual representation of these mechanisms, the textual elucidation provides a comprehensive insight into the micro-level interactions among XC polymer, PAC-LV, and starch. The study reveals that the equilibrium achieved by these components within Spacer A results in a spacer fluid with optimal performance characteristics. The intricate balance of gel resistance, plastic viscosity, and fluid loss control is crucial for the successful application of Spacer A in drilling operations. Despite the lack of visual aids, the textual description offers readers a thorough comprehension of how the molecular interplay among these admixtures governs the macroscopic rheological behavior of Spacer A. This approach aligns with the study's objective to deepen the understanding of designed spacer fluids and their effectiveness in practical drilling scenarios.

The rotor test is conducted to evaluate the ability of one fluid to clean another fluid. In this study, the rotor test was performed to measure the ability of spacer fluid to clean drilling fluid from the metal wall of a cylinder. The test involves separating the metal cylinder from the rod and weighing it dry (W1). The weight of the metal cylinder in the dry state is constant at 125/519 gr. Next, the cylinder is connected to the rod and placed in the drilling fluid container. The mixer is turned on at 2000 RPM, and the cylinder rotates in the drilling fluid for 10 min. After turning off the device, the cylinder is removed and allowed to dry for 1 min before being weighed (W2) and recorded. The same procedure is repeated with spacer fluid, and the cylinder is weighed again (W3) after being removed and dried for 1 min. This process is repeated for other fluids, and the results are recorded. Using equation 23-3 and the three weights, the efficiency of spacer fluid in cleaning drilling fluid from the metal cylinder wall can be determined by equation (11) ([15]; Hussein et al. , 2020).

(11) $$\%\text{Mud}\;\text{Removal}=\frac{W_{2}-W_{3}}{W_{2}-W_{1}}$$

### 3.2 The effect of designed spacers on mud removal

The purpose of this study was to investigate the effect of designed spacers on mud cleaning using the rotor test. Two cases were examined: case 1, where the fluids were tested normally, and case 2, where Sodium dodecyl sulfate (SDS) surfactant was added to the fluids to evaluate its effect on mud removal. In case 1, spacer A showed no significant change in mud removal rate with changes in additive concentration. Spacer B had better purification than other fluids at lower concentrations of starch and PAC-LV, but this ability was lost with increasing additive concentration. Spacer C was similar to spacer B, and increasing starch concentration significantly reduced the fluid's ability to remove mud. In case 2, SDS surfactant was added to the spacer at concentrations of 0.25% and 0.50% by weight, and the effect of adding this surfactant on the fluids can be seen in the following diagram.

### 3.3 Optimizing the concentration of fluid lost circulation materials

**Fig. 18:**
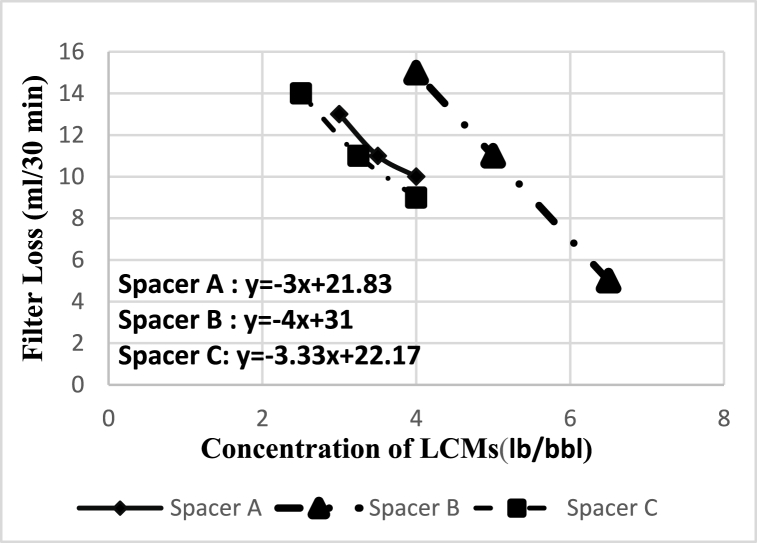
The effect of concentration of different additives on the filter loss of the spacer and their linear equation.

The test results of the 9 spacer models designed are shown in Table 9. Fig. 18 shows the filter loss results for the spacer A, B and C. In general, with increasing the weight of the spacer, the flow rate of its filter loss also increases, which is due to the increase in the concentration of solid particles in the fluid. Also, the thickness of the mud cake increases with increasing flow rate of filter loss.

According to the results of Table 9 and the linear equation of the relationship between the concentration of additives and the filter loss (Fig. 18), it is found that the slope of the spacer equation B is higher than the other two spacers and the presence of starch and PAC-LV together, it has further reduced the amount volume of filtrate. Subsequently, the slope of the spacer C equation was greater than that of spacer A, indicating that starch had a greater effect on the control of spacer than PAC-LV, but overall the filter loss in all three spacers was acceptable. And at higher concentrations than the values used, the viscosity of these fluids increases and it becomes more difficult to pump these fluids into the well.

**Table 9:** Effect of concentration of LCMs on loss of spacer fluid in filter press test.

Sample	A_1_	A_2_	A_3_	B_1_	B_2_	B_3_	C_1_	C_2_	C_3_
concentration of LCMs (lb/bbl)	**3**	**3.5**	**4**	**4**	**5**	**6.5**	**2.5**	**3.25**	**4**
Filter Loss volume(ml)	**13**	**11**	**10**	**15**	**11**	**5**	**14**	**11**	**9**

### 3.4 Evaluation of compatibility of spacer rheology with drilling fluid and cement slurry

**Fig. 19:**
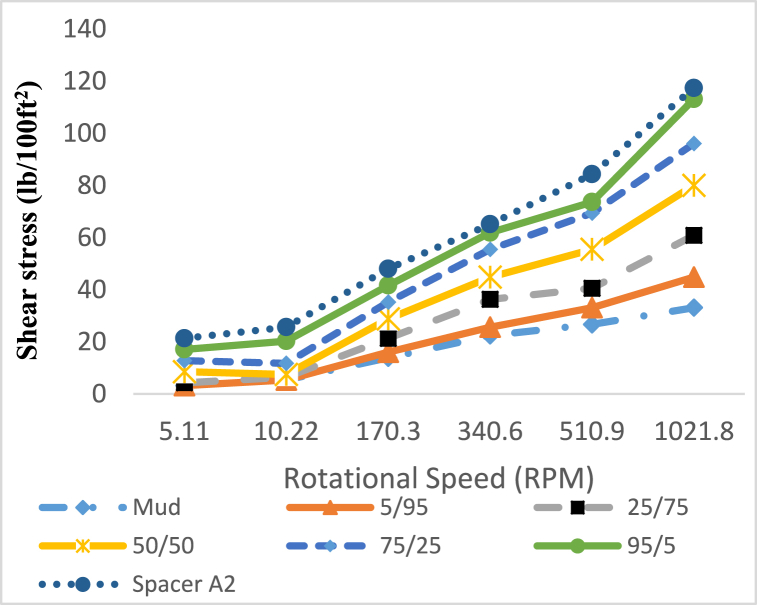
Rheological profile of spacer A2 (79 pcf) mixed with WBS at various spacer to drilling fluid ratios in volume at 25 °C.

**Fig. 20:**
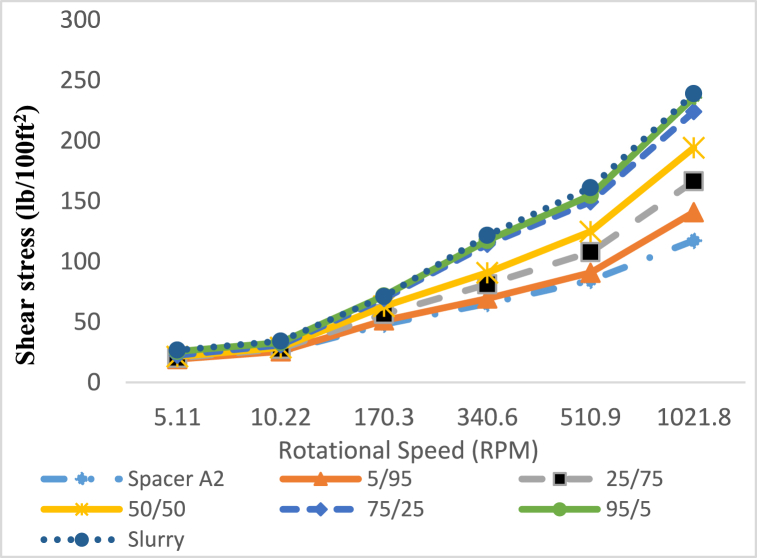
Rheological profile of spacer A2 (79 pcf) mixed with Cement Slurry at various spacer to drilling fluid ratios in volume at 25 °C.

**Fig. 21:**
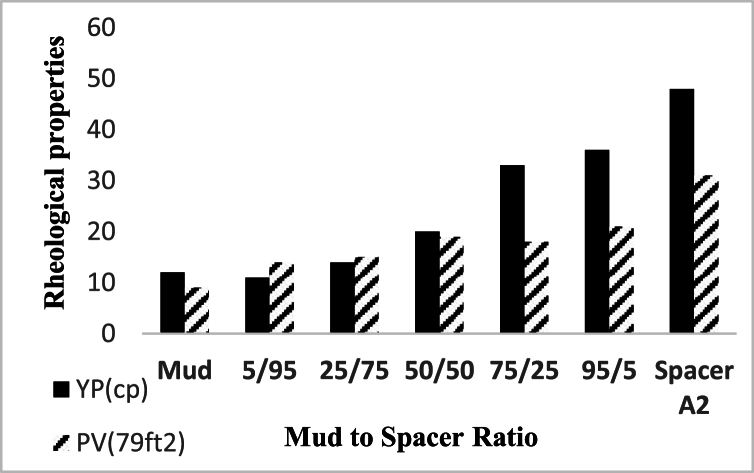
Rheological properties of spacer A2 (79 pcf) mixed with WBS at various spacer to drilling fluid ratios in volume at 25 °C.

**Fig. 22:**
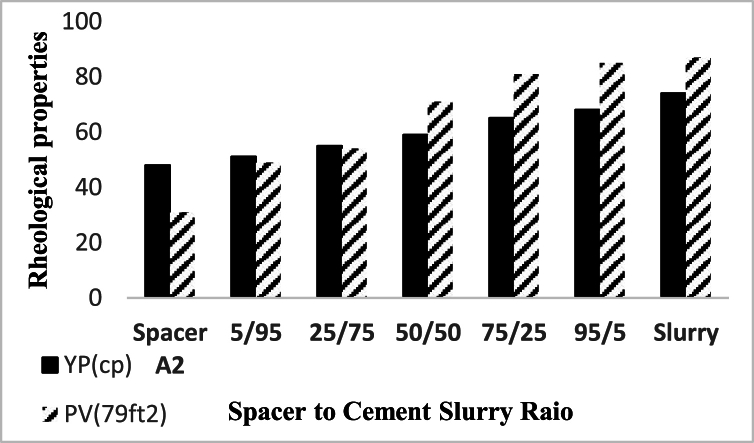
Rheological properties of spacer A2 (79 pcf) mixed with Cement Slurry at various spacer to drilling fluid ratios in volume at 25 °C.

In case of incompatibility between the spacer fluid and the drilling fluid or the cement slurry, changes in the rheological properties of the fluids occur when they come into contact with each other. In order to check the compatibility of fluids and prevent adverse events caused by the incompatibility of wellbore fluids, it is necessary to investigate the rheological properties of these fluids in contact with each other in different volume ratios. Rheological properties between compatible fluids are proportional to the volume ratio of the mixture of two compatible fluids. For example, plastic viscosity is a rheological property that for a mixture of cement slurry and spacer fluid with a ratio of 50/50, the numerical value of this property is between its value for both fluids in pure form. In fluid compatibility test, drilling fluid with a density of 71 pcf, spacer with a density of 80 pcf (Spacer A2 was selected as a sample) and cement slurry with a density of 114 pcf were used. Fig. 19, Fig. 20 show the results of shear stress changes with different shear rates according to different proportions of these tested fluids have been shown, and no inconsistency was observed in the obtained results. Moreover, Fig. 21, Fig. 22 specifically detail the rheological properties of spacer A2 (79 pcf) mixed with WBS and Cement Slurry, respectively, at varying volume ratios and the same temperature.

The micro mechanisms underlying the performance of the three spacers (Spacer A, Spacer B, and Spacer C) can be elucidated by examining their compositions and rheological properties. Spacer A comprises XC polymer, PAC-LV, and starch, which collectively define its distinct characteristics. The XC Polymer within Spacer A forms elongated polymer chains and a wide lattice structure, resulting in heightened gel resistance and an associated increase in plastic viscosity. PAC-LV, particularly when combined with starch, further enhances gel resistance, contributing to a heightened level of viscosity. While Starch within Spacer A contributes to gel resistance, its impact is overshadowed by XC and PAC-LV, though it plays a significant role in controlling fluid loss.

In contrast, Spacer B lacks XC polymer but incorporates starch and PAC-LV. The micro mechanisms within Spacer B involve the collaborative action of PAC-LV and Starch, working together to regulate fluid loss and provide gel resistance. Starch assumes a potentially more dominant role in controlling fluid loss due to the absence of XC Polymer. The absence of XC Polymer in Spacer B results in a lower gel resistance compared to Spacer A, leading to a reduction in plastic viscosity.

Spacer C, with its composition encompassing XC polymer, PAC-LV, and starch, exhibits micro mechanisms akin to Spacer A. The XC Polymer in Spacer C contributes to heightened gel resistance and increased plastic viscosity. PAC-LV and Starch within Spacer C operate synergistically to regulate fluid loss and provide additional gel resistance. The well-balanced combination of these components in Spacer C achieves acceptable fluid loss and viscosity, demonstrating its effectiveness as a spacer fluid.

Overall, the micro mechanisms involve the intricate interplay of XC polymer, PAC-LV, and starch at varying concentrations. XC Polymer emerges as pivotal for achieving heightened gel resistance and plastic viscosity, while PAC-LV and starch play crucial roles in fluid loss control and gel resistance. The nuanced effects of these components, influenced by the overall composition of each spacer, collectively determine the rheological and fluid loss properties of Spacer A, Spacer B, and Spacer C.

The economic significance of this study lies in its potential to optimize drilling operations, leading to cost savings and increased efficiency in the oil and gas industry. The tailored design and comprehensive evaluation of spacer fluids, particularly Spacer A, offer the industry a targeted solution for enhancing drilling fluid performance. By understanding the intricate rheological behavior and micro mechanisms of spacer fluids, operators can make informed decisions to improve drilling efficiency, reduce downtime, and minimize costs associated with fluid-related challenges. The ability to control fluid loss, maintain wellbore stability, and enhance cleaning efficiency directly impacts the overall success of drilling operations. Implementing optimized spacer fluids, as informed by this study, can contribute to significant economic benefits by mitigating issues related to wellbore instability, equipment wear, and overall drilling fluid performance. Moreover, the study's focus on compatibility with other drilling fluids and cement slurries ensures a holistic approach to wellbore management, reducing the risk of complications during drilling and completion processes. Overall, the economic importance of this work lies in its potential to streamline drilling operations, improve efficiency, and reduce costs through the strategic utilization of well-designed spacer fluids. Table 10 provides a simplified comparison between a conventional spacer fluid and the optimized spacer fluid (Spacer A) in various aspects related to economic considerations in drilling operations. The actual values or ratings may vary based on specific conditions and the outcomes of the study.

**Table 10:** Economic comparison between conventional spacer fluid and optimized spacer fluid (spacer A) in drilling operations.

Aspect	Conventional Spacer Fluid	Optimized Spacer Fluid (Spacer A)
Fluid-related Downtime	High	Reduced
Equipment Wear and Tear	Moderate to High	Reduced
Drilling Efficiency	Standard	Enhanced
Fluid-related Costs	Moderate to High	Potentially Reduced
Cleaning Efficiency	Standard	Improved
Compatibility Issues	Occasional	Addressed
Overall Drilling Cost	Significant	Potential Reduction
Wellbore Stability	Varies	Improved
Fluid Loss Control	Standard	Enhanced

## 4 Conclusion

In conclusion, this study provides comprehensive insights into the critical aspects of spacer fluid composition and rheological modeling tailored for drilling applications. The designed spacers—Spacer A, Spacer B, and Spacer C—underwent systematic evaluation, with a specific emphasis on the influence of XC polymer, PAC-LV, and starch on rheological behavior. The Herschel-Buckley model emerged as the most accurate in describing spacer fluid behavior. Practical assessments, including the rotor test and mud removal test, unequivocally identify Spacer B1 as the most efficient mud remover. Incorporating 0.51% PAC-LV and 0.51% starch, Spacer B1 demonstrated an impressive 67.84% mud removal rate, reinforcing the findings stated in the abstract. This assertion is explicitly reiterated in the conclusion, affirming Spacer B1's status as the most successful mud remover with a numerical value, thus addressing the discrepancy pointed out in the comment. Furthermore, the addition of Sodium dodecyl sulfate (SDS) surfactant showcased its impact on cleaning efficiency, illustrating the adaptability of spacer fluids. Fluid loss control tests underscored Spacer B's effectiveness in managing fluid loss, evident from the steeper slope in the concentration-filter loss relationship. Compatibility tests confirmed satisfactory rheological compatibility with drilling fluid and cement slurry, a critical factor in maintaining wellbore stability. An in-depth exploration of Spacer A's micro mechanisms highlighted the intricate interplay of XC polymer, PAC-LV, and starch, contributing to a well-balanced rheological profile. Although visual plots were omitted, the textual elucidation aimed to offer profound insights into how molecular interactions govern Spacer A's macroscopic rheological behavior, enriching the overall comprehension of spacer fluids' rheological properties and their practical application in drilling scenarios. While this study provides valuable insights into the rheological behavior and performance of the designed spacer fluids, certain limitations need acknowledgment. The experiments were conducted under controlled laboratory conditions, necessitating caution when extrapolating findings to diverse field conditions affected by temperature variations, pressure fluctuations, and other subsurface conditions. Additionally, the primary focus on Spacer A limits the direct applicability of the findings to other spacer compositions. To address these limitations, future research should involve field trials under real-world drilling conditions and explore a broader range of spacer compositions for a more generalized understanding of identified mechanisms. Collaborative efforts with industry partners could facilitate on-site testing, providing comprehensive data for practical applications. Despite these limitations, this study lays a solid foundation for further exploration and refinement of spacer fluid formulations in drilling operations.

## Data availability statement

Every one wants the data can emails the Corrosponding Author.

## CRediT authorship contribution statement

**Amanj Salimi:** Writing – review & editing, Writing – original draft, Methodology, Investigation, Formal analysis, Data curation. **Ali Heidari Beni:** Writing – original draft, Methodology, Investigation, Formal analysis, Data curation. **Mohammad Bazvand:** Writing – review & editing, Writing – original draft, Project administration, Methodology, Investigation, Formal analysis, Data curation, Conceptualization.

## Declaration of competing interest

The authors declare the following financial interests/personal relationships which may be considered as potential competing interests:The financial support of this work has been done by the Faculty of Oil and Gas, 10.13039/501100011702Sahand University of Technology, Tabriz, Iran.
